# Supplementary material for: A systematic review of quantum machine learning for digital health
Source: NPJ Digit Med. 2025 May 2;8:237. doi: 10.1038/s41746-025-01597-z (PMC12048600; doi:10.1038/s41746-025-01597-z)
Supplement: Supplementary file 1 — Supplementary Information [file 41746_2025_1597_MOESM1_ESM.pdf]

Supplementary Note 1

We briefly introduce notation and terminology associated with quantum algorithms. Quantum states are represented by the notation of a ket $|\cdot\rangle$. We will consider a specific type of quantum state, called a qubit (or quantum bit). At the extremal points, a qubit occupies the state associated classical binary bit $|0\rangle, |1\rangle$. However, a qubit can also occupy values that are superposition states,

$$|\psi\rangle := \alpha|0\rangle + \beta|1\rangle, \tag{1}$$

where α, β are complex amplitudes satisfying $|\alpha|^2 + |\beta|^2 = 1$, and the linear combination of $|0\rangle, |1\rangle$ has a well-defined relative phase. Indeed, the relative phase relationships between quantum states in superposition allows quantum states to interfere, i.e. add or cancel out, in ways that may enhance computations. When measured, quantum systems are also inherently probabilistic, i.e. in the above example, the measured qubit will be associated with a binary outcome 0 (1) with probability $|\alpha|^2$ ($|\beta|^2$). For most applications, these quantum measurements are repeated for N_{shots} number of times in order to build a statistical distribution over measurement outcomes from which useful quantities are inferred.

We can demystify quantum computations by thinking of them as basic operations in linear algebra. Here, kets are represented as column vectors in a complex linear space, and quantum operations are represented by matrices, which act on states via matrix multiplication. For every quantum state ket, we associate a bra, $\langle\psi|$, which can be thought of as a row vector containing the complex conjugate of elements of $|\psi\rangle$. To compare any two quantum states, $|\psi\rangle$ and $|\phi\rangle$, we compute their overlap as an inner product between $\langle\phi|$ and $|\psi\rangle$, denoted $\langle\phi|\psi\rangle$. To summarize quantum algorithms, it is useful to represent quantum states with matrices as well as vectors. The matrix representation of a quantum state $|\psi\rangle$ is the density matrix, $\rho := |\psi\rangle\langle\psi|$, such that ρ is an outer product. The density operator is required to satisfy three mathematical properties: unit trace ($\mathrm{Tr}[\rho] = 1$), hermicity ($\rho = \rho^\dagger$) and positive semi-definiteness. These requirements ensure that measurement statistics can always be associated to quantum probability amplitudes (e.g. α, β) of an underlying quantum state. In the above, mathematical operations are denoted by the trace Tr, $\langle\cdot\rangle$ denotes an inner product, and † denotes complex conjugation.

Nearly all transformations of a quantum state, including those within quantum machine learning algorithms in the presence of realistic noise, are called quantum channels. These channels, denoted η, can represent transformations such as quantum gates (rotations), quantum measurements, or the impact of noise on a quantum state interacting with its environment. For all these phenomena, we represent channels as,

$$\eta(\rho) := \sum_i K_i \rho K_i^\dagger, \tag{2}$$

where K_i represents a quantum gate, measurement, or noise process. For the transformation $\eta(\rho)$ to output a physically valid quantum state, η must satisfy mathematical properties (completely positivity and $0 \leq \mathrm{Tr}[\eta(\rho)] \leq 1$) or alternatively, $\sum_i K_i^\dagger K_i \leq 1$.

Quantum circuits only represent a simple subset of all possible quantum channels. By placing restrictions on what η can represent, we can draw η as a quantum circuit. A quantum circuit contains horizontal wires representing qubits where input states are shown as ket symbols to the left of each wire and temporal order progresses from left to right. Boxed symbols represent quantum gates or quantum measurements on relevant wires. The main restriction is that boxed quantum gates must be reversible rotations of quantum states, i.e. unitary operations satisfying $K^\dagger = K^{-1}$. With measurements pushed to the end, a quantum circuit typically represents a unitary quantum channel with only one term in the sum in equation (2), i.e. $i \in \{1\}$, and therefore only an ensemble of quantum circuits can represent the collective action of noisy quantum circuits. The term 'circuit size' represents the number of qubits, while 'circuit depth' represents the number of time steps required to run the full circuit. Circuit depth assumes that quantum operations have been parallelized where possible and therefore refers to the minimum number of sequential steps, rather than a full tally of quantum gates in a circuit. While measurements are typically ignored until the very end, some measurements are performed 'mid-circuit'. If these mid-circuit measurement outcomes, or measured qubits, are not used in subsequent processing, then they can be safely pushed to the end of the circuit. In other cases, a double-wire may be used to visually represent how outcomes of mid-circuit measurements can be used to change quantum operations 'on the fly'.

Extracting information from a quantum computer is inherently statistical. In the case of qubits, all quantum measurements form a distribution of random '0' or '1' outcomes i.e. binary outcomes of a Bernoulli trial. Typically there is some desired physical quantity, $\hat{O}$, whose average value must be computed from this distribution of quantum measurements. The quantum state ρ is prepared, and the type (or basis) of quantum measurements is chosen according to $\hat{O}$. The statistical average (expectation value) of $\langle \hat{O} \rangle$ is then represented by,

$$f := \text{Tr}[\rho \hat{O}], \tag{3}$$

where f is estimated empirically by collecting experimental data on a quantum computer using N_{shots}, and the right-hand side of the equation is a mathematical description of the information extraction process. Since $\text{Tr}[A^\dagger B] = \langle B, A \rangle$ represents an inner product, f is some measure of the overlap between our quantum state ρ and the desired operation $\hat{O}$, analogous to the overlap of quantum states represented as vectors. Indeed if $\rho = |\psi\rangle\langle\psi|$, then $\text{Tr}[\rho \hat{O}] = \text{Tr}[|\psi\rangle\langle\psi|\hat{O}] = \text{Tr}[\langle\psi|\hat{O}|\psi\rangle] = \langle\psi|\hat{O}|\psi\rangle$ which is a scalar number representing the average value of $\hat{O}$ under $|\psi\rangle$. Computing this average requires repeatedly preparing $|\psi\rangle$, measuring this quantum circuit in the appropriate basis N_{shots} number of times, and using classical post-processing of distribution of measurement outcomes. All quantum algorithms in this review extract information from quantum computers in this manner.

Let $f(x, \theta)$ be the average output of a quantum algorithm, and ρ_0 be the input quantum state, e.g. where all qubits are in their ground (zero) state, and where (x, θ) define classical inputs to a quantum algorithm. Here, x represents one sample of real data with dimension d, $x \in \mathbb{R}^d$, for a dataset containing a total of N data samples. Meanwhile, we also define tunable free parameters, θ, that potentially could implement tunable quantum gates. The desired output information required from the algorithm is typically given by $\hat{O}$. We now discuss specific quantum algorithms that prepare quantum states, transform these states, and extract information from quantum computers in order to complete learning tasks.

Quantum neural networks (QNNs) consist of input data (x-dependent) and tunable (θ-dependent) quantum operations. Generally, the output of a QNN is,

$$f(x, \theta) := \text{Tr}\left[U(x, \theta)\rho_0 U^\dagger(x, \theta)\hat{O}\right] = \langle \rho_{x,\theta}, \hat{O} \rangle, \tag{4}$$

where the data (x-dependent) and tunable (θ-dependent) components of QNNs cannot be separated. In the above, $U(x, \theta)$ represents a parameterized quantum gate which depends on data x and tunable parameters θ. The equation above computes the overlap between information in the quantum state $\rho_{x,\theta} = U(x, \theta)\rho_0 U^\dagger(x, \theta)$, and the desired output $\hat{O}$, using an inner product.

In contrast, linear quantum models allow us to separate the x-dependent quantum operations and θ-dependent quantum operations within the inner product [1]. In these models, we perform data encoding operations followed by tunable gates $V(\theta)$. As shown in Figure 1(a) in the main text, a linear QNN can be expressed by,

$$f(x, \theta) := \text{Tr}\left[U(x)\rho_0 U^\dagger(x) V^\dagger(\theta)\hat{O}V(\theta)\right] = \langle \rho_x, \hat{O}_\theta \rangle \tag{5}$$

In the above, θ can take the form of any other classical parameters that are not x, data encoding is expressed by $\rho_x := U(x)\rho_0 U^\dagger(x)$, and the parameterized neural net is expressed as $\hat{O}_\theta := V^\dagger(\theta)\hat{O}V(\theta)$.

With this structure, we can describe many quantum machine learning algorithms. For example, we can omit θ entirely, and recover sophisticated algorithms that focus on data encoding procedures. In kernel methods, θ is replaced by training data, and the algorithm output f during prediction represents a linear combination of all training samples. Sometimes the action of ρ, $U(x)$ or $V(\theta)$ is non-trivially restricted to some subset of quantum states, yielding so-called quantum transformers. These choices are valid examples of linear quantum models, discussed below.

Quantum kernel methods (QKMs) are expressed as linear quantum models by replacing free tunable parameters θ by optimized linear combinations of training data given by $(\alpha, \mathcal{X}_T)$. To see this, we redefine the second term in the inner product, $\hat{O}_\theta \equiv \hat{O}_{\alpha,\mathcal{X}_T} := \sum_{t=1}^{T} \alpha_t \rho(x_t)$ for training data $x_t \in \mathcal{X}_T, t =$

$1, \ldots, T$. Substituting this expression into the inner product, the output of quantum kernel methods is

$$f(x, \alpha, x_t) := \sum_{t=1}^{T} \alpha_t \langle \rho_x, \rho_{x_t} \rangle, \tag{6}$$

where the inner product compares the overlap between two quantum states parameterized by two data points, x and training data sample, x_t. The weights, α, are optimized during training.

Quantum transformers, such as those of synthesized studies in Cherrat (2024) and Landman (2022), use sophisticated data encoders and neural network structures. Here, the data loaders ensure that the encoded state ρ_x consists of all possible combinations of states where all but one qubit is nonzero, i.e. states with Hamming weight-1 like '00010' or '10000' but not '10100'. The action of the quantum neural network is then chosen to ensure that output superpositions of quantum states are also of Hamming weight of one. In particular, let ρ_x denote the Hamming weight-1 inputs, Λ represents the restriction of linear algebra operations that preserve the weight of these states, and V_Λ the quantum operation which implements $V_\Lambda|x\rangle = |\Lambda x\rangle$. Here, some choice of weight matrix Λ enables one to compute linear multiplication Λx using a quantum circuit. Choosing $\hat{O}_\Lambda \equiv V_\Lambda \hat{O} V_\Lambda$ yields all the data loaders introduced in both Cherrat (2024) and Landman (2022). Similarly, one can add a trainable layer with parameters θ such that a quantum operation implements a trainable matrix multiplication, $V_W(\theta)|x\rangle = |W(\theta)x\rangle$, where trainability of W is made explicit in notation. For one of the algorithms, the quantum orthogonal transformer discussed in Cherrat (2024), we find that the output function for computing the so-called attention mechanism $A_{i,j}$ for two data patches x_i, x_j, is

$$A_{i,j} \equiv f(x_i, \theta, x_j) := \mathrm{Tr}\left[U^\dagger(x_j) V_W(\theta) U(x_i) \rho_0 U^\dagger(x_i) V_W^\dagger(\theta) U(x_j) \hat{O}\right] = \langle \rho_{x_i}, \hat{O}_{x_j,\theta} \rangle, \tag{7}$$

$$\hat{O}_{x_j,\theta} = V_W^\dagger(\theta) U(x_j) \hat{O} U^\dagger(x_j) V_W(\theta). \tag{8}$$

In the above, the term $\hat{O}_{x_j,\theta}$ is reminiscent of a quantum kernel method since it depends on another data sample $x_j \neq x_i$, but also depends on a trainable matrix $W(\theta)$ that affects parameterization of quantum gates. Assuming two different patches, $x_i \neq x_j$, one can factorize the inner product by grouping x_j, θ, and argue that the inner product remains linear in x_i. Unlike kernel methods, however, x_j is not limited to the training dataset and consists of all pairwise combinations in the data. Indeed if $x_i \equiv x_j$, then the output function would be non-linear in x_i in a manner similar to non-linear quantum models [1] such as quantum data re-uploading classifiers (QDRCs) depicted for reference in Figure 1(c) in the main text [2].

Quantum convolutional neural networks (QCNNs) can similarly be understood as a tunable quantum channel $\tilde{\eta}_\theta$ that is composed of many smaller channels η_{θ_i}, where quantum channels are introduced earlier. For each i-th tunable layer, the algorithm's structure can be written as $\tilde{\eta}_\theta(\rho) := \bigcirc_i \eta_{\theta_i}(\rho) = \ldots \eta_{\theta_3}(\eta_{\theta_2}(\eta_{\theta_1}(\rho))) \ldots$. In typical formulations such as that in the main text in Figure 1(d), in each i-th layer, we measure half of the remaining qubits in that layer, forcing these measured qubits to be reduced to classical bits. Consequently, these channels η_{θ_i} are defined on an increasingly smaller number of qubits as i increases, until only one qubit is left. The channels for each layer η_{θ_i} are typically non-unitary, meaning that unlike quantum gates, these operations cannot be reversed or 'undone'. An example of a non-unitary channel is where the measurement outcomes of a pooling layer dictate how gates are applied to the remaining qubits in the next layer [3]. If we want to extract $f(x, \theta) = \mathrm{Tr}\left[\tilde{\eta}_\theta(\rho_x)\hat{O}\right]$, then in general it appears that tunable parameters and data-dependent operations cannot be separated for QCNNs. However, if mid-circuit QCNN measurements do not affect future quantum operations and can all be safely pushed to the end of the QCNN circuit, then averaging over mid-circuit measurements can be implemented entirely in classical post-processing and the layers η_{θ_i} can be unitary (i.e. $\eta_{\theta_i}(\rho) := V(\theta_i)\rho V^\dagger(\theta_i)$). In this regime, we recover a linear quantum model for QCNNs,

$$f(x, \theta) = \mathrm{Tr}_{i \neq q}\left[\rho_x \tilde{\eta}_\theta^\dagger(\hat{O})\right], \tag{9}$$

where the trace over i conveys that all pooling layers are marginalized at the end except for the last remaining qubit, q. There is some heuristic evidence that linear QCNN models of this form are unlikely to be useful for the analysis of classical data [4].

Quantum causal modeling is the use of quantum algorithms to solve causal inference problems. In medical settings, establishing causality between variables based on real-world medical data is an important classical learning task. If the direction of a certain causal relationship is known, then the causal effect, which represents the strength of the causal relationship, can be estimated via classical or quantum techniques. In this review, the quantum techniques used for causal inference were all linear quantum models of the form above.

Quantum deep reinforcement learning networks rely on the same underlying capabilities of a quantum neural network and the specific examples encountered in this review fit into the framework of linear quantum models. The only modification is the introduction of reinforcement learning, whereby a classical learning agent is trained to take optimal actions in a given environment to maximize a pre-determined reward function. Since the learning agent is classical, the underlying role of quantum technologies is the same as any other linear quantum model.

Quantum generative adversarial networks use the interaction between two artificial learning agents that have access to a quantum computer. The generator and discriminator are adversarial: the generator creates random, synthetic data samples with the goal of fooling the discriminator into believing it is real data, while the discriminator must assign a label of real or fake to each data sample. The end point of this game is a generator that has been trained to create high quality synthetic samples such that the discriminator is forced to guess randomly, i.e. discriminator guesses correctly with 50% probability. In the quantum versions of these algorithms, the data refers to quantum states of a quantum system, the generator has access to a quantum computer, and the discriminator can perform arbitrary quantum measurements. QGANs may demonstrate exponential quantum advantage for sufficiently high-dimensional quantum data [5]. For classical datasets, no provable quantum advantage exists. The quantum circuits formed by QGANs in this review were also linear quantum models.

Supplementary Note 2

PRISMA 2020 for Abstracts Checklist

Section and Topic	Item #	Checklist item	Reported (Yes/No)
**TITLE**			
Title	1	Identify the report as a systematic review.	Yes
**BACKGROUND**			
Objectives	2	Provide an explicit statement of the main objective(s) or question(s) the review addresses.	Yes
**METHODS**			
Eligibility criteria	3	Specify the inclusion and exclusion criteria for the review.	Yes
Information sources	4	Specify the information sources (e.g. databases, registers) used to identify studies and the date when each was last searched.	Yes
Risk of bias	5	Specify the methods used to assess risk of bias in the included studies.	NA
Synthesis of results	6	Specify the methods used to present and synthesise results.	Yes
**RESULTS**			
Included studies	7	Give the total number of included studies and participants and summarise relevant characteristics of studies.	Yes
Synthesis of results	8	Present results for main outcomes, preferably indicating the number of included studies and participants for each. If meta-analysis was done, report the summary estimate and confidence/credible interval. If comparing groups, indicate the direction of the effect (i.e. which group is favoured).	Yes
**DISCUSSION**			
Limitations of evidence	9	Provide a brief summary of the limitations of the evidence included in the review (e.g. study risk of bias, inconsistency and imprecision).	Yes
Interpretation	10	Provide a general interpretation of the results and important implications.	Yes
**OTHER**			
Funding	11	Specify the primary source of funding for the review.	NA
Registration	12	Provide the register name and registration number.	NA

PRISMA 2020 Checklist

Section and Topic	Item #	Checklist item	Location where item is reported
**TITLE**			
Title	1	Identify the report as a systematic review.	Title
**ABSTRACT**			
Abstract	2	See the PRISMA 2020 for Abstracts checklist.	Abstract
**INTRODUCTION**			
Rationale	3	Describe the rationale for the review in the context of existing knowledge.	S1
Objectives	4	Provide an explicit statement of the objective(s) or question(s) the review addresses.	S2
**METHODS**			
Eligibility criteria	5	Specify the inclusion and exclusion criteria for the review and how studies were grouped for the syntheses.	S3
Information sources	6	Specify all databases, registers, websites, organisations, reference lists and other sources searched or consulted to identify studies. Specify the date when each source was last searched or consulted.	S3 Appendix
Search strategy	7	Present the full search strategies for all databases, registers and websites, including any filters and limits used.	Appendix
Selection process	8	Specify the methods used to decide whether a study met the inclusion criteria of the review, including how many reviewers screened each record and each report retrieved, whether they worked independently, and if applicable, details of automation tools used in the process.	S3 Appendix
Data collection process	9	Specify the methods used to collect data from reports, including how many reviewers collected data from each report, whether they worked independently, any processes for obtaining or confirming data from study investigators, and if applicable, details of automation tools used in the process.	S3
Data items	10a	List and define all outcomes for which data were sought. Specify whether all results that were compatible with each outcome domain in each study were sought (e.g. for all measures, time points, analyses), and if not, the methods used to decide which results to collect.	S3
	10b	List and define all other variables for which data were sought (e.g. participant and intervention characteristics, funding sources). Describe any assumptions made about any missing or unclear information.	S3
Study risk of bias assessment	11	Specify the methods used to assess risk of bias in the included studies, including details of the tool(s) used, how many reviewers assessed each study and whether they worked independently, and if applicable, details of automation tools used in the process.	NA
Effect measures	12	Specify for each outcome the effect measure(s) (e.g. risk ratio, mean difference) used in the synthesis or presentation of results.	S3, S4.1
Synthesis methods	13a	Describe the processes used to decide which studies were eligible for each synthesis (e.g. tabulating the study intervention characteristics and comparing against the planned groups for each synthesis (item #5)).	S4.1
	13b	Describe any methods required to prepare the data for presentation or synthesis, such as handling of missing summary statistics, or data conversions.	S4.1
	13c	Describe any methods used to tabulate or visually display results of individual studies and syntheses.	S4.1
	13d	Describe any methods used to synthesize results and provide a rationale for the choice(s). If meta-analysis was performed, describe the model(s), method(s) to identify the presence and extent of statistical heterogeneity, and software package(s) used.	S4.1
	13e	Describe any methods used to explore possible causes of heterogeneity among study results (e.g. subgroup analysis, meta-regression).	S4.1
	13f	Describe any sensitivity analyses conducted to assess robustness of the synthesized results.	NA
Reporting bias assessment	14	Describe any methods used to assess risk of bias due to missing results in a synthesis (arising from reporting biases).	NA
Certainty assessment	15	Describe any methods used to assess certainty (or confidence) in the body of evidence for an outcome.	S3, S4.1

PRISMA 2020 Checklist

Section and Topic	Item #	Checklist item	Location where item is reported
**RESULTS**			
Study selection	16a	Describe the results of the search and selection process, from the number of records identified in the search to the number of studies included in the review, ideally using a flow diagram.	S4.1
	16b	Cite studies that might appear to meet the inclusion criteria, but which were excluded, and explain why they were excluded.	S4.1
Study characteristics	17	Cite each included study and present its characteristics.	S4.1
Risk of bias in studies	18	Present assessments of risk of bias for each included study.	NA
Results of individual studies	19	For all outcomes, present, for each study: (a) summary statistics for each group (where appropriate) and (b) an effect estimate and its precision (e.g. confidence/credible interval), ideally using structured tables or plots.	S4.1, S4.2
Results of syntheses	20a	For each synthesis, briefly summarise the characteristics and risk of bias among contributing studies.	S4.2
	20b	Present results of all statistical syntheses conducted. If meta-analysis was done, present for each the summary estimate and its precision (e.g. confidence/credible interval) and measures of statistical heterogeneity. If comparing groups, describe the direction of the effect.	S4.2
	20c	Present results of all investigations of possible causes of heterogeneity among study results.	S4.2
	20d	Present results of all sensitivity analyses conducted to assess the robustness of the synthesized results.	NA
Reporting biases	21	Present assessments of risk of bias due to missing results (arising from reporting biases) for each synthesis assessed.	NA
Certainty of evidence	22	Present assessments of certainty (or confidence) in the body of evidence for each outcome assessed.	S4.2
**DISCUSSION**			
Discussion	23a	Provide a general interpretation of the results in the context of other evidence.	S5
	23b	Discuss any limitations of the evidence included in the review.	S5
	23c	Discuss any limitations of the review processes used.	S5
	23d	Discuss implications of the results for practice, policy, and future research.	S5
**OTHER INFORMATION**			
Registration and protocol	24a	Provide registration information for the review, including register name and registration number, or state that the review was not registered.	S3
	24b	Indicate where the review protocol can be accessed, or state that a protocol was not prepared.	Declarations
	24c	Describe and explain any amendments to information provided at registration or in the protocol.	Declarations
Support	25	Describe sources of financial or non-financial support for the review, and the role of the funders or sponsors in the review.	Declarations
Competing interests	26	Declare any competing interests of review authors.	Declarations
Availability of data, code and other materials	27	Report which of the following are publicly available and where they can be found: template data collection forms; data extracted from included studies; data used for all analyses; analytic code; any other materials used in the review.	Declarations

Supplementary Note 3

For sixteen synthesized studies, we report available open-source code and datasets, where available with NR./UR. indicating Not Reported / Upon Request.

Synthesized Study	Ref.	Code	Data
Nyugen 2018	[6]	NR./UR.	Synthetic
Piat 2018	[7]	NR./UR.	Private
Yano 2020	[8]	NR./UR.	UCI MLR Heart Disease Data.<br>UCI Wisconsin Breast Cancer Data.
Niraula 2021	[9]	NR./UR.	Private: RTOG0617 dataset collected under NCT00533949-D1 (c.f. NCTN)
Krunic 2022	[10]	NR./UR.	Private: Optum de-identified EHR dataset
Landman 2022	[11]	NR./UR.	MEDMINST Data.
Moradi 2022	[12]	Git repo	UCI Pediatric Bone Marrow transplant Data.<br>UCI Wisconsin Breast Cancer Data.<br>Kaggle Heart Failure Data.
Das 2023	[13]	NR./UR.	Private: MRIs of human ear collected in laboratory
Guddanti 2023	[14]	Git repo	Kaggle Pneumonia Chest X Ray Data
Kawaguchi 2023	[15]	NR./UR.	Kaggle Pima Indian Diabetes Data.<br>UCI MLR Heart Disease Data.
Moradi 2023	[16]	Git repo	Papp L et. al. Data. Grahovac M et. al. Data. Papp L, Pötsch N et al. Data.
Qu 2023	[17]	Git repo	MIT-BIH Arrhythmia & ST Change, European ST-T, Sudden Cardiac Death.
Asiwga 2024	[18]	NR./UR.	UCI Wisconsin Breast Cancer Data.
Cherrat 2024	[19]	NR./UR.	MEDMINST Data.
Kazdaghli 2024	[20]	Git repo	Synthetic, EHR MIMIC-III Data.
Choi 2024	[21]	Git repo	Private survey datasets.

Supplementary Note 4

Abbrev.	Definition
EHR	Electronic health records
EMR	Electronic medical records
GAN	Generative adversarial network
LDA	Linear discriminant analysis
PCA	Principal component analysis
PRISMA	Preferred reporting items for systematic reviews and meta-Analyses
PROSPERO	International prospective register of systematic reviews
PTRI	Phase space terrain ruggedness index, a proposed global metric for linking performance scores with a configuration space [10]
PQC	Parameterized quantum circuit
QC	Quantum computation
QCNN	Quantum convolutional neural network algorithms
QEC	Quantum error correction
QEM	Quantum error mitigation
QGAN	Quantum generative adversarial methods
QKM	Quantum kernel methods
QPIE	Quantum probability image encoding [22]
QML	Quantum machine learning algorithms
QNN	Quantum neural network algorithms
QRAC	Quantum random access coding of bitstring length m into n qubits so that any 1 out of m bits can be recovered with probability $p > 1/2$
QRAM	Quantum random access memory
QUBO	Quadratic unconstrained binary optimization
SVM	Support vector machine
VQC	Variational quantum circuit

Supplementary Note 5

Search strategies for databases are provided below. While PubMed and Embase are popular in health, IEEE and Scopus typically index computer science, physics and engineering journals. Physics-centric publications, e.g. APS journals and Quantum, are indexed in Scopus, while quantum physics preprint server arXiv (quant-ph) captures recent and unindexed literature.

Database	Setting/Perspective	Intervention	Comparator	Eval / Study Characteristics
arXiv	health<br>OR clinical<br>OR "medical data"<br>OR "medical record"<br>OR “medical imaging”<br>OR “medical image”<br>OR hospital<br>OR "patient data"<br>OR "radiography"<br>OR "ECG"<br>OR "EEG"<br>OR "MRI"<br>OR "electrocardiography"<br>OR "uss"<br>OR "ct"<br>OR ultrasound*<br>OR "echography"<br>OR "x-ray"<br>OR "x ray"<br>OR "xray"<br>OR mammogr*<br>OR "electroencephalography"<br>OR "laboratory data"<br>OR "laboratory test"<br>OR biostatistics<br>OR radiology<br>OR epiproteom*<br>OR epiomic*<br>OR vaccin*<br>OR epigenom*<br>OR metabolom*<br>OR gene<br>OR transcriptom*<br>OR proteom*<br>OR genom*	Quantum<br><br>All Fields	"machine learning"<br>OR algorithm*<br>OR Bayes*<br>OR kernel*<br>OR classif*<br>OR "neural net"<br>OR "reinforcement learning"<br>OR "k-means"<br>OR "wavelet"<br>OR "support vector"<br>OR "random forest"<br>OR "Boltzmann machine"<br>OR "adversarial net"<br>OR "random walk"<br>OR "regression"<br>OR "monte carlo"<br>OR "Markov"<br><br>All Fields	
PubMed	"medical records"[MeSH Terms]<br>OR "electronic health records"[MeSH Terms]<br>OR "electronic health record*"[All Fields]<br>OR "health record*"[All Fields]<br>OR "medical record*"[All Fields]<br>OR "clinical record*"[All Fields]<br>OR ("hospital"[All Fields] AND "data*"[All Fields])<br>OR ("clinical"[All Fields] AND "data*"[All Fields])<br>OR ("medical"[All Fields] AND "data*"[All Fields])<br>OR ("health"[All Fields] AND "data*"[All Fields])<br>OR ("patient*"[All Fields] AND "data*"[All Fields])<br>OR “medical history”<br>OR "Routinely Collected Health Data"[Mesh]<br>OR "Routinely Collected Health Data"[All Fields]<br>OR "Administrative Data"[All Fields]<br>OR "Administrative Health Data" [All Fields]<br>OR "health information systems"[MeSH Terms]<br>OR "health services administration"[MeSH Terms]<br>OR "medical informatics computing"[MeSH Terms]<br>OR "medical inform*"[All Fields]<br>OR "radiography"[MeSH Terms]<br>OR "radiography"[All Fields]<br>OR ("medical"[All Fields] AND "imaging"[All Fields])<br>OR "medical imaging"[All Fields]<br>OR "diagnostic imaging"[MeSH Terms]<br>OR ("diagnostic"[All Fields] AND "imaging"[All Fields])<br>OR "diagnostic imaging"[All Fields]<br>OR "radiography"[MeSH Terms]<br>OR "electrocardiography"[MeSH Terms]<br>OR "ecg"[All Fields]<br>OR "electrocardiography"[MeSH Terms]<br>OR "ekg"[All Fields]<br>OR “radiograph*”<br>OR "magnetic resonance imaging"[MeSH Terms]<br>OR ("magnetic"[All Fields] AND "resonance"[All Fields])<br>OR "magnetic resonance imaging"[All Fields]<br>OR "mri"[All Fields]<br>OR " nuclear magnetic resonance imaging”	“quantum machine learning”[All Fields]<br>OR “quantum comput*”[All Fields]<br>OR “quantum inform*”[All Fields]<br>OR quantum[Title/Abstract]	(machine learning[MeSH Terms])<br>OR “machine learning”<br>OR algorithm[MeSH Terms]<br>OR “algorithm*”[All Fields]<br>OR “Bayes*”[All Fields]<br>OR “kernel*”[All Fields]<br>OR “classif*”[All Fields]<br>OR "neural networks, computer"[MeSH Terms]<br>OR "deep learning"[MeSH Terms]<br>OR (cnn)<br>OR (svm)<br>OR (svd)<br>OR “pca”[Title/Abstract]<br>OR “principal component analysis”[All Fields]<br>OR “reinforcement learning”[All Fields]<br>OR “k-means”[All Fields]<br>OR “wavelet”[All Fields]<br>OR “genetic algorithm*”[All Fields]<br>OR “neural net*”[All Fields]<br>OR “support vector*”[All Fields]<br>OR “random forest*”[All Fields]<br>OR “Boltzmann mach*”[All Fields]<br>OR “adversarial net*”[All Fields]<br>OR “random walk*”[All Fields]<br>OR “linear regression”[All Fields]<br>OR “nonlinear regression”[All Fields]<br>OR "monte carlo method"[MeSH Terms]<br>OR "Markov chain”[All Fields]<br>OR “gaussian process regression”[All Fields]<br>OR “ChatGPT”<br>OR “large language models”[All Fields]<br>OR “artificial intelligence”[All Fields]<br>OR “big data” [All Fields]<br>OR “data pre-processing”[All Fields]<br>OR “data post-processing”[All Fields]<br>OR “feature selection”[All Fields]<br>OR “feature extraction”[All Fields]<br>OR “predictive model*”[All Fields]	("2015"[Date - Publication] : "3000"[Date - Publication])<br><br>NOT ("systematic review"[pt] OR "meta-analysis"[pt] OR "review"[pt] OR "clinical trial protocol"[pt] OR "clinical conference"[pt] OR "case reports"[pt])

	OR "coherence tomography"<br>OR “optical coherence tomography”<br>OR “computer assisted tomography”<br>OR "uss"[All Fields]<br>OR "ct"[All Fields]<br>OR "computed tomography"[All Fields]<br>OR “ultrasound*”<br>OR “echography”<br>OR "x rays"[MeSH Terms]<br>OR "x rays"[All Fields]<br>OR "xray"[All Fields]<br>OR “mammogra*”<br>OR “holography”<br>OR "electroencephalography"[MeSH Terms]<br>OR "electroencephalography"[All Fields]<br>OR "eeg"[All Fields]<br>OR “radiology”[Title/Abstract]<br>OR “laboratory data”<br>OR “laboratory test*”<br>OR “biostatistics”[Title/Abstract]<br>OR epigenomics[Mesh]<br>OR metabolomics[Mesh]<br>OR biomarker[Mesh]<br>OR transcriptomics[Mesh]<br>OR proteomics[Mesh]<br>OR genomics[Mesh]<br>OR “epiproteom*”<br>OR “epiomic*”<br>OR “vaccin*”<br>OR “epigenom*”<br>OR “metabolom*”<br>OR “gene”<br>OR “transcriptom*”<br>OR “proteom*”<br>OR “genom*”			
Embase (Elservier)	'medical record'/exp<br>OR 'electronic health record*'<br>OR 'health record*'<br>OR 'medical record*'<br>OR 'clinical record*'<br>OR (hospital AND data*)<br>OR (clinical AND data*)<br>OR (medical AND data*)<br>OR (health AND data*)<br>OR (patient* AND data*)<br>OR 'medical history'<br>OR 'Routinely Collected Health Data'/exp<br>OR 'Routinely Collected Health Data'<br>OR 'Administrative Data'<br>OR 'Administrative Health Data'<br>OR 'medical information system'/exp<br>OR 'medical information'/exp<br>OR 'medical inform*'<br>OR radiography<br>OR (medical AND imaging)<br>OR 'medical imaging'<br>OR 'diagnostic imaging'/exp<br>OR (diagnostic AND imaging)<br>OR 'diagnostic imaging'<br>OR radiography/exp<br>OR electrocardiography/exp<br>OR ecg<br>OR electrocardiography/exp<br>OR ekg<br>OR radiograph*<br>OR 'nuclear magnetic resonance imaging'/exp<br>OR (magnetic AND resonance)<br>OR 'magnetic resonance imaging'<br>OR mri<br>OR 'nuclear magnetic resonance imaging'<br>OR 'coherence tomography'<br>OR 'optical coherence tomography'<br>OR 'computer assisted tomography'<br>OR uss<br>OR ct<br>OR 'computed tomography'<br>OR ultrasound*<br>OR echography<br>OR 'X ray flim'/exp<br>OR 'x rays'<br>OR xray<br>OR mammogra*<br>OR holography<br>OR electroencephalography/exp<br>OR electroencephalography<br>OR eeg<br>OR radiology:ti,ab<br>OR 'laboratory data'	'quantum machine learning'<br>OR 'quantum comput*'<br>OR 'quantum inform*'<br>OR quantum:ti,ab	('machine learning'/exp)<br>OR 'machine learning'<br>OR algorithm/exp<br>OR algorithm*<br>OR Bayes*<br>OR kernel*<br>OR classif*<br>OR (cnn)<br>OR (svm)<br>OR (svd)<br>OR (pca):ti,ab<br>OR 'principal component analysis'<br>OR 'reinforcement learning'<br>OR k-means<br>OR wavelet<br>OR 'genetic algorithm*'<br>OR 'neural net*'<br>OR 'support vector*'<br>OR 'random forest*'<br>OR 'Boltzmann mach*'<br>OR 'adversarial net*'<br>OR 'random walk*'<br>OR 'linear regression'<br>OR 'nonlinear regression'<br>OR 'monte carlo’<br>OR 'Markov chain'<br>OR 'gaussian process regression'<br>OR ChatGPT<br>OR 'large language models'<br>OR 'artificial intelligence'<br>OR 'big data'<br>OR 'data pre-processing'<br>OR 'data post-processing'<br>OR 'feature selection'<br>OR 'feature extraction'<br>OR 'predictive model*'	NOT ('conference abstract'/it OR 'review'/it) AND [2015-2024]/py

	OR 'laboratory test*'<br>OR biostatistics:ti,ab<br>OR epigenomics/exp<br>OR metabolomics/exp<br>OR biomarker/exp<br>OR transcriptomics/exp<br>OR proteomics/exp<br>OR genomics/exp<br>OR epiproteom*<br>OR epiomic*<br>OR vaccin*<br>OR epigenom*<br>OR metabolom*<br>OR gene<br>OR transcriptom*<br>OR proteom*<br>OR genom*			
Scopus	INDEXTERMS("medical records")<br>OR INDEXTERMS("electronic health records")<br>OR ALL("electronic health record*")<br>OR ALL("health record*")<br>OR ALL("medical record*")<br>OR ALL("clinical record*")<br>OR (ALL(hospital) AND ALL(data*))<br>OR (ALL(clinical) AND ALL(data*))<br>OR (ALL(medical) AND ALL(data*))<br>OR (ALL(health) AND ALL(data*))<br>OR (ALL(patient*) AND ALL(data*))<br>OR "medical history"<br>OR INDEXTERMS("Routinely Collected Health Data")<br>OR ALL("Routinely Collected Health Data")<br>OR ALL("Administrative Data")<br>OR ALL("Administrative Health Data")<br>OR INDEXTERMS("health information systems")<br>OR INDEXTERMS("health services administration")<br>OR INDEXTERMS("medical informatics computing")<br>OR ALL("medical inform*")<br>OR INDEXTERMS(radiography)<br>OR ALL(radiography)<br>OR (ALL(medical) AND ALL(imaging))<br>OR ALL("medical imaging")<br>OR INDEXTERMS("diagnostic imaging")<br>OR (ALL(diagnostic) AND ALL(imaging))<br>OR ALL("diagnostic imaging")<br>OR INDEXTERMS(radiography)<br>OR INDEXTERMS(electrocardiography)<br>OR ALL(ecg)<br>OR INDEXTERMS(electrocardiography)<br>OR ALL(ekg)<br>OR radiograph*<br>OR INDEXTERMS("magnetic resonance imaging")<br>OR (ALL(magnetic) AND ALL(resonance))<br>OR ALL("magnetic resonance imaging")<br>OR ALL(mri)<br>OR "nuclear magnetic resonance imaging"<br>OR "coherence tomography"<br>OR "optical coherence tomography"<br>OR "computer assisted tomography"<br>OR ALL(uss)<br>OR ALL(ct)<br>OR ALL("computed tomography")<br>OR “ultrasound*”<br>OR “echography”<br>OR INDEXTERMS("x rays")<br>OR ALL("x rays")<br>OR ALL(“xray”)<br>OR “mammogra*”<br>OR “holography”<br>OR INDEXTERMS(electroencephalography)<br>OR ALL(electroencephalography)<br>OR ALL(eeg)<br>OR TITLE-ABS(radiology)<br>OR "laboratory data"<br>OR "laboratory test*"<br>OR TITLE-ABS(biostatistics)<br>OR INDEXTERMS(epigenomics)<br>OR INDEXTERMS(metabolomics)<br>OR INDEXTERMS(biomarker)<br>OR INDEXTERMS(transcriptomics)<br>OR INDEXTERMS(proteomics)<br>OR INDEXTERMS(genomics)<br>OR “epiproteom*”	ALL("quantum machine learning")<br>OR ALL("quantum comput*")<br>OR ALL("quantum inform*")<br>OR TITLE-ABS(quantum)	INDEXTERMS("machine learning")<br>OR "machine learning"<br>OR INDEXTERMS(algorithm)<br>OR ALL(algorithm*)<br>OR ALL(Bayes*)<br>OR ALL(kernel*)<br>OR ALL(classif*)<br>OR INDEXTERMS("neural networks, computer")<br>OR INDEXTERMS("deep learning")<br>OR (cnn)<br>OR (svm)<br>OR (svd)<br>OR TITLE-ABS(pca)<br>OR ALL("principal component analysis")<br>OR ALL("reinforcement learning")<br>OR ALL(k-means)<br>OR ALL(wavelet)<br>OR ALL("genetic algorithm*")<br>OR ALL("neural net*")<br>OR ALL("support vector*")<br>OR ALL("random forest*")<br>OR ALL("Boltzmann mach*")<br>OR ALL("adversarial net*")<br>OR ALL("random walk*")<br>OR ALL("linear regression")<br>OR ALL("nonlinear regression")<br>OR ALL("Markov carlo")<br>OR ALL("Markov chain")<br>OR ALL("gaussian process regression")<br>OR ChatGPT<br>OR ALL("large language models")<br>OR ALL("artificial intelligence")<br>OR ALL("big data")<br>OR ALL("data pre-processing")<br>OR ALL("data post-processing")<br>OR ALL("feature selection")<br>OR ALL("feature extraction")<br>OR ALL("predictive model*")	2015 ><br><br>Exclude: Reviews, Conference Reviews, Books, Book Chapters

	OR “epiomic*”<br>OR “vaccin*”<br>OR “epigenom*”<br>OR “metabolom*”<br>OR “gene”<br>OR “transcriptom*”<br>OR “proteom*”<br>OR “genom*”			
IEEE	"Mesh_Terms":"medical record"<br>OR "electronic health record?"<br>OR (("hospital" OR "clinical" OR "medical" OR "health" OR "patient") AND "record?")<br>OR (("hospital" OR "clinical" OR "medical" OR "health" OR "patient") AND "data*")<br>OR "medical history"<br>OR "Mesh_Terms":"Routinely Collected Health Data"<br>OR "Routinely Collected Health Data"<br>OR "Administrative Data"<br>OR "Administrative Health Data"<br>OR "Mesh_Terms":"medical information system"<br>OR "Mesh_Terms":"medical information"<br>OR "medical inform*"<br>OR "radiography"<br>OR ("medical" AND "imaging")<br>OR "medical imaging"<br>OR "Mesh_Terms":"diagnostic imaging"<br>OR ("diagnostic" AND "imaging")<br>OR "diagnostic imaging"<br>OR "Mesh_Terms":"radiography"<br>OR "Mesh_Terms":"electrocardiography"<br>OR "ecg"<br>OR "Mesh_Terms":"electrocardiography"<br>OR "ekg"<br>OR "radiograph*"<br>OR "Mesh_Terms":"nuclear magnetic resonance imaging"<br>OR ("magnetic" AND "resonance")<br>OR "magnetic resonance imaging"<br>OR mri<br>OR "nuclear magnetic resonance imaging"<br>OR "coherence tomography"<br>OR "optical coherence tomography"<br>OR "computer assisted tomography"<br>OR uss<br>OR ct<br>OR "computed tomography"<br>OR "ultrasound?"<br>OR "echography"<br>OR "Mesh_Terms":"X ray flim"<br>OR "x rays"<br>OR "xray"<br>OR "mammogra*"<br>OR "holography"<br>OR "Mesh_Terms":"electroencephalography"<br>OR "electroencephalography"<br>OR "eeg"<br>OR "Document Title":"radiology"<br>OR "Abstract":"radiology"<br>OR "laboratory data"<br>OR "laboratory test*"<br>OR "Document Title":"biostatistics"<br>OR "Abstract":"biostatistics"<br>OR "Mesh_Terms":"epigenomics”<br>OR "Mesh_Terms":"metabolomics”<br>OR "Mesh_Terms":"biomarker”<br>OR "Mesh_Terms":"transcriptomics”<br>OR "Mesh_Terms":"proteomics”<br>OR "Mesh_Terms":"genomics”<br>OR “epiproteom*”<br>OR “epiomic*”<br>OR “vaccin*”<br>OR “epigenom*”<br>OR “metabolom*”<br>OR “gene”<br>OR “transcriptom*”<br>OR “proteom*”<br>OR “genom*”	"quantum machine learning"<br>OR "quantum comput*"<br>OR "quantum inform*"<br>OR "Document Title":"quantum"<br>OR "Abstract":"quantum"	("machine learning")<br>OR "Mesh_Terms":"machine learning"<br>OR "Mesh_Terms":"algorithm"<br>OR "algorithm?"<br>OR ("Bayes" OR "Bayesian")<br>OR "kernel?"<br>OR ("classifier" OR "classification")<br>OR ("cnn")<br>OR ("svm")<br>OR ("svd")<br>OR "Document Title":"pca"<br>OR "Abstract":"pca"<br>OR "principal component analysis"<br>OR "reinforcement learning"<br>OR "k-means"<br>OR "wavelet"<br>OR "genetic" ORNEAR/2 ("algorithm?")<br>OR "neural" ORNEAR/2 ("network?" OR "net?")<br>OR "support vector?"<br>OR "random forest?"<br>OR "Boltzmann" ONEAR/3 "machine"<br>OR "adversarial" ONEAR/3 ("network?" OR "net?")<br>OR "random walk?"<br>OR "linear regression"<br>OR "nonlinear regression"<br>OR "monte carlo’<br>OR "Markov chain"<br>OR "gaussian process regression"<br>OR "ChatGPT"<br>OR "large language models"<br>OR "artificial intelligence"<br>OR "big data"<br>OR "data pre-processing"<br>OR "data post-processing"<br>OR "feature selection"<br>OR "feature extraction"<br>OR "predictive" ORNEAR/2 ("model?" OR "modelling")	Manual

Supplementary Note 6

The data extraction template is provided below.

	Field name	Type	Details
1	Title	Free text	Title of the article
2	Year	Free text	Publication year
3	Country	Country	Country of first author affiliation
4	Input health dataset(s)	Multi-select; Free text "Other"	Medical images: MRIs<br>Medical images: CT<br>Medical images: XRays<br>Medical images: thermal<br>Medical images: ultrasound<br>Time varying signals: EEG<br>Time varying signals: ECG<br>Time varying signals: heart rate<br>Medical data: patient demographics<br>Medical data: laboratory tests<br>Medical data: other (specify)<br>Textual or handwritten medical data<br>Other
5	Input data is sourced from	Multi-select; Free text "Other"	Open sourced or public datasets<br>Privately sourced datasets<br>EHR / EM or hospital records<br>Data collected during study<br>Other
6	QML intervention(s):	Multi-select; Free text "Other"	Quantum kernels<br>Quantum neural networks (QNN)<br>Quantum convolutional neural net (QCNN)<br>Quanvolutional neural net<br>Quantum long short time memory (QLSTM)<br>Quantum support vector machine<br>Quantum PCA<br>Quantum Fourier transform<br>Grover search<br>HHL algorithm<br>Quantum transfer learning<br>Variational/parameterized quantum circuits<br>Quantum genetic / evolutionary<br>Quantum adversarial network<br>Q K-means / clustering<br>Other
7	Classical comparator(s)	Multi-select; Free text "Other"	Support vector machines<br>Principal component analysis<br>Classical kernels<br>Regression<br>Tensor networks<br>Bayesian analysis<br>Neural networks (NN)<br>Convolutional neural networks (CNN)<br>Genetic or evolutionary algorithms<br>Gaussian process regression<br>Nonlinear or particle filters<br>Other
8	Simulators for quantum algorithms	Multi-select; Free text "Other"	Pennylane<br>Cirq<br>Qiskit<br>IBM Lab / IBM Composer<br>MATLAB<br>Mathematica<br>Other
9	Experimental data collection on hardware	Multi-select; Free text "Other	None used / not applicable<br>IBM Falcon<br>IBM Heron<br>IBM Eagle<br>Older IBM device<br>Quantinuum H1<br>Quantinuum H2<br>Google Sycamore<br>Google Bristlcone<br>Rigetti<br>Xanadu<br>Psi-Quantum<br>Other
10	Approx circuit depth	Free text	NR: Not reported; NA : Not applicable
11	Approx circuit size (e.g. qubit number)	Free text	NR: Not reported; NA : Not applicable
12	Code and data is accessible	Boolean	Yes/ No<br>Not sufficient to provide link to public datasets. Must provide access to codebase via Github or other public link
13	Q1: Explains quantum algorithm selection by referencing learning problem class or dataset structure:	Single choice	0 : No theory or empirical rationale for quantum algorithm selection discussed or cited<br>1 : Quantum algorithm selection is empirical or mostly cites empirical literature<br>2 : Quantum algorithm selection is linked to underlying class of learning problem or data structure<br>3 : Quantum algorithm has provable advantage with respect to class of learning problem or data structure
14	Q2: Identifies/ discusses impact of data encoding on quantum algorithm performance:	Single choice	0 : Encoding methodology omitted or incomplete<br>0 : Impact of different encoding strategies on overall performance is not analyzed<br>1 : Performance impact is discussed with incomplete analysis (e.g. compares at least 2 methods)<br>2 : Performance impact is well characterized empirically or theoretically
15	Q3: Identifies/ discusses impact of classical input data processing on quantum algorithm performance:	Single choice	0 : Data pre-processing methodology is omitted or incomplete<br>0 : Impact of different data pre-processing strategies on overall performance is not analyzed<br>1 : Performance impact is discussed with incomplete analysis (e.g. compares at least 2 methods)<br>2 : Performance impact is well characterized empirically or theoretically<br>Not applicable (no classical input data processing)
16	Q4: (EMPIRICAL ONLY) Dimensionality of data input for quantum algorithm	Single choice	0 : Not reported or discussed; or unclear<br>0 : Negligible i.e. O(1)<br>1 : Small i.e. O(10)<br>2 : Intermediate i.e. O(10^2)<br>3 : Large i.e. O(10^3) or greater<br>Not applicable (theory study)
17	Judgement	Single choice	Do not proceed if at least two scores from Q1, Q2, Q3, or Q4 is zero.<br>EXCLUDE STUDY<br>CONTINUE EXTRACTION
18	Any judgement comments?	Free text	
19	Sample size	Single choice	This refers to the sample size of data processed by a quantum algorithm e.g. 256 images.<br>0: Applicable but not reported<br>0: Negligible O(1)<br>1: Small i.e. O(10^1)<br>2 : Medium i.e. O(10^2)<br>3: Large i.e. O(10^3)<br>4: XLarge i.e. O(10^4) or greater<br>Not applicable (theory study)
20	QML and classical algorithms received input data of same dimensionality	Single choice,	True<br>False<br>Not described / unknown
21	QML and classical algorithms received training data of same sample size	Single choice	True<br>False<br>Not described / unknown
22	Loss function	Single choice; free text "Other"	Not applicable<br>Not described / unknown<br>Lp norm<br>Other
23	Regularization	Single choice, free text "Other"	Not applicable<br>Not described / unknown<br>Explicitly regularized<br>Other
24	Hyperparameter tuning	free text "Other"	Not applicable<br>Not described / unknown / not tuned<br>Known apriori initialization conditions<br>Manually tuned / ad hoc<br>Grid-search<br>Optimization via meta-protocol<br>Other
25	Discussion of realistic operating conditions:	Multi-select	Shot noise<br>Noise: depolarizing, bit flip or amplitude<br>Noise: : Pauli channels<br>Noise: Hardware noise sources<br>Data noise: Missing data<br>Data noise: Incorrect training data<br>Model generalization error<br>Model optimization: hyper parameter tuning<br>Model optimization: loss landscape<br>Hardware execution: number of qubits<br>Hardware execution: layout / connectivity<br>Hardware execution: gates, msmts, control<br>Clinical efficacy / implementation<br>Other
26	Discussion of realistic operating conditions:	Single choice	0 : None of the above operating conditions discussed<br>1 : Between 1 to 3 operating conditions discussed (without analysis)<br>2 : Between 1 to 3 operating conditions discussed with at least one with quantitative analysis<br>3 : More than 3 operating conditions discussed with quantitative analysis
27	Simulators (ML)	Multi-select	None reported<br>Python Llibrary: TensorFlow<br>Python library: scikitlearn<br>Python library: other<br>NVIDIA CPU/GPU simulator<br>Julia library<br>MATLAB<br>Mathematica<br>C++ libraries<br>Google Colab<br>Other
28	Risk of bias assessment	Boolean	Not applicable<br>Applicable
29	For synthesis?	Boolean	Must be INCLUDED for full extraction<br>Must use hardware or discuss / simulate the affect of noise

Supplementary References

[1] S. Jerbi, L.J. Fiderer, H. Poulsen Nautrup, J.M. Kübler, H.J. Briegel, V. Dunjko, Quantum machine learning beyond kernel methods. Nat. Commun. **14**(517), 1–8 (2023). https://doi.org/10.1038/s41467-023-36159-y

[2] A. Pérez-Salinas, A. Cervera-Lierta, E. Gil-Fuster, J.I. Latorre, Data re-uploading for a universal quantum classifier. Quantum **4**, 226 (2020). https://doi.org/10.22331/q-2020-02-06-226. 1907.02085v2

[3] I. Cong, S. Choi, M.D. Lukin, Quantum convolutional neural networks. Nat. Phys. **15**, 1273–1278 (2019). https://doi.org/10.1038/s41567-019-0648-8

[4] P. Bermejo, P. Braccia, M.S. Rudolph, Z. Holmes, L. Cincio, M. Cerezo, Quantum Convolutional Neural Networks are (Effectively) Classically Simulable. arXiv (2024). https://doi.org/10.48550/arXiv.2408.12739. 2408.12739

[5] S. Lloyd, C. Weedbrook, Quantum generative adversarial learning. Physical Review Letters **121**(4) (2018). https://doi.org/10.1103/PhysRevLett.121.040502. Cited by: 327; All Open Access, Green Open Access

[6] N.T.T. Nguyen, G.T. Kenyon, *Radiographic Inference Based on a Model of V1 Simple Cells Implemented on the D-Wave 2X Quantum Annealing Computer*, in *2018 IEEE International Conference on Rebooting Computing (ICRC)*. pp. 1–6. https://doi.org/10.1109/ICRC.2018.8638621. URL https://ieeexplore.ieee.org/document/8638621/

[7] S. Piat, N. Usher, S. Severini, M. Herbster, T. Mansi, P. Mountney, Image classification with quantum pre-training and auto-encoders. International Journal of Quantum Information **16**(8) (2018). https://doi.org/10.1142/S0219749918400099

[8] H. Yano, Y. Suzuki, R. Raymond, N. Yamamoto, *Efficient Discrete Feature Encoding for Variational Quantum Classifier* (Institute of Electrical and Electronics Engineers Inc.), pp. 11–21. https://doi.org/10.1109/QCE49297.2020.00012

[9] D. Niraula, J. Jamaluddin, M.M. Matuszak, R.K.T. Haken, I.E. Naqa, Quantum deep reinforcement learning for clinical decision support in oncology: application to adaptive radiotherapy. Scientific Reports **11**(1) (2021). https://doi.org/10.1038/s41598-021-02910-y. Cited By :13 Export Date: 3 June 2024

[10] Z. Krunic, F. Flother, G. Seegan, N. Earnest-Noble, S. Omar, Quantum kernels for real-world predictions based on electronic health records. IEEE Transactions on Quantum Engineering **3** (2022). https://doi.org/10.1109/TQE.2022.3176806

[11] J. Landman, N. Mathur, Y.Y. Li, M. Strahm, S. Kazdaghli, A. Prakash, I. Kerenidis, Quantum methods for neural networks and application to medical image classification. Quantum **6** (2022). https://doi.org/10.22331/Q-2022-12-22-881

[12] S. Moradi, C. Brandner, C. Spielvogel, D. Krajnc, S. Hillmich, R. Wille, W. Drexler, L. Papp, Clinical data classification with noisy intermediate scale quantum computers. Scientific Reports **12**(1) (2022). https://doi.org/10.1038/s41598-022-05971-9

[13] S. Das, J. Zhang, S. Martina, D. Suter, F. Caruso, Quantum pattern recognition on real quantum processing units. Quantum Machine Intelligence **5**(1) (2023). https://doi.org/10.1007/s42484-022-00093-x

[14] S.S. Guddanti, A. Padhye, A. Prabhakar, S. Tayur, Pneumonia detection by binary classification: classical, quantum, and hybrid approaches for support vector machine (svm). Frontiers in Computer Science **5** (2023). https://doi.org/10.3389/fcomp.2023.1286657

[15] H. Kawaguchi, Application of quantum computing to a linear non-gaussian acyclic model for novel medical knowledge discovery. PLoS ONE **18**(4 April) (2023). https://doi.org/10.1371/journal.pone.0283933

[16] S. Moradi, C. Spielvogel, D. Krajnc, C. Brandner, S. Hillmich, R. Wille, T. Traub-Weidinger, X. Li, M. Hacker, W. Drexler, L. Papp, Error mitigation enables pet radiomic cancer characterization on quantum computers. European Journal of Nuclear Medicine and Molecular Imaging **50**(13), 3826–3837 (2023). https://doi.org/10.1007/s00259-023-06362-6

[17] Z. Qu, W. Shi, P. Tiwari, Quantum conditional generative adversarial network based on patch method for abnormal electrocardiogram generation. Computers in Biology and Medicine **166** (2023). https://doi.org/10.1016/j.compbiomed.2023.107549

[18] R.V. Aswiga, S. Sridevi, B. Indira, Leveraging quantum kernel support vector machine for breast cancer diagnosis from digital breast tomosynthesis images. Quantum Machine Intelligence **6**(2) (2024). https://doi.org/10.1007/s42484-024-00170-3

[19] E.A. Cherrat, I. Kerenidis, N. Mathur, J. Landman, M. Strahm, Y.Y. Li, Quantum vision transformers. Quantum **8**, 1265 (2024). https://doi.org/10.22331/q-2024-02-22-1265

[20] S. Kazdaghli, I. Kerenidis, J. Kieckbusch, P. Teare, Improved clinical data imputation via classical and quantum determinantal point processes. Elife **12** (2024). https://doi.org/10.7554/eLife.89947

[21] J. Choi, K. Kim, S.H. Park, J. Hur, H. Yang, Y.H. Kim, H. Lee, S. Han, Investigation of factors regarding the effects of covid-19 pandemic on college students' depression by quantum annealer. Scientific Reports **14**(1) (2024). https://doi.org/10.1038/s41598-024-54533-8. URL https://www.scopus.com/inward/record.uri?eid=2-s2.0-85185929353&doi=10.1038%2fs41598-024-54533-8&partnerID=40&md5=f20d2fe0279a4ebd249138c437f330f7https://www.nature.com/articles/s41598-024-54533-8.pdf

[22] X.W. Yao, H. Wang, Z. Liao, M.C. Chen, J. Pan, J. Li, K. Zhang, X. Lin, Z. Wang, Z. Luo, W. Zheng, J. Li, M. Zhao, X. Peng, D. Suter, Quantum Image Processing and Its Application to Edge Detection: Theory and Experiment. arXiv (2018). https://doi.org/10.1103/PhysRevX.7.031041. 1801.01465
